# Effects of seasonal temperature variability on glucose levels in acute ischemic stroke patients

**DOI:** 10.1007/s00484-025-03103-2

**Published:** 2026-01-05

**Authors:** Wei Miaomiao, Wang Fuyin, Xia Xiaoshuang, Wang Lin, Li Xin

**Affiliations:** 1 Department of Neurology, The Second Hospital of Tianjin Medical University, No.23, PingJiang Road, Tianjin, 300211 China; 2 Department of Geriatrics, The Second Hospital of Tianjin Medical University, Tianjin, China; 3 Tianjin Interdisciplinary Innovation Centre for Health and Meteorology, Tianjin, China

**Keywords:** Acute ischemic stroke, Blood glucose parameters, Seasonal temperature variability, Temperature effect, Clinical severity

## Abstract

This study investigated seasonal temperature variability in glycemic parameters and their clinical correlates in 8730 acute ischemic stroke (AIS) patients admitted from 2013 to 2022. Patients were grouped by season of onset, with collection of demographic characteristics, laboratory results, admission NIHSS scores and meteorological data. After confounder adjustment, generalized additive model(GAM) was used to evaluate the correlation between ambient temperature, glycemic parameters and NIHSS scores, with interactions examined via season stratification. In patients with diabetes, fasting plasma glucose (FPG) levels in winter increased by 1.22 mmol/l (95% *CI*:1.14,1.29 mmol/l, *P* < 0.01) versus summer. General linear model analysis revealed lower FPG levels in summer (*β* = -0.083, 95% *CI*: -0.726, -0.062; *P* < 0.05) and autumn (*β* = -0.125, 95% *CI*: -0.590, -0.073; *P* < 0.05) versus winter. HbA1c demonstrated similar seasonal variation, particularly when values exceeding 7.0%, with spring-autumn differences reaching 0.81 units (95% *CI*: 0.61, 1.02; *P* < 0.05). HbA1c variations were most pronounced in spring (*β* = 0.201, 95% *CI*: 0.099, 0.557; *P* < 0.001) and summer (*β* = 0.107, 95% *CI*: 0.096, 0.449; *P* < 0.05) versus autumn. Winter admissions correlated with greater stroke severity, supported by 1 ◦C temperature decrease was associated with 2.5 (95% *CI*: -4.5, -0.5;*P* < 0.01) NIHSS scores increase. Of note, each 0.1 mmol/L increment in FPG corresponding to a 0.8 rise in NIHSS scores (95% *CI*: 0.5, 1.1, *P* < 0.01), while HbA1c showed no association. Our findings demonstrated AIS patients showed seasonal glucose fluctuations, with the highest hyperglycemia and severity in winter. The robust association between FPG and outcomes highlighted the need for seasonally-adjusted glucose management in high-risk populations.

**Supplementary Information:**

The online version contains supplementary material available at 10.1007/s00484-025-03103-2.

## Introduction

As one of the major worldwide causes of death and disability, ischemic stroke is proven to be of seasonal variation across diverse populations and geographical regions (Kurtz et al. 2021; Fujii et al. 2021; Xue et al. 2023). Several trigger factors have been proposed to explain the seasonality in vascular diseases, such as acute infections, hypercoagulable state and increases in blood pressure due to low ambient temperature, as well as seasonal fluctuations in serum lipids and glucose level (Narita and Kario 2023; Huta-Osiecka et al. 2021). Seasonal variables have been evaluated for their impact on cerebrovascular risk factors, with winter months consistently associated with adverse changes in physiological parameters and risk profiles. Substantial evidence supports the seasonal variation of fasting plasma glucose (FPG), with peak levels typically observed during colder months (Honda et al. 2021; Vallianou et al. 2021). This phenomenon appears physiologically linked to the acute effects of ambient temperature, as previous studies have demonstrated a negative relationship between temperature and FPG levels (Luo et al. 2021; He et al. 2022). Given that elevated FPG levels are associated with an increased risk of stroke (Bian et al. 2025), these periodic glucose metabolism shifts may contribute to the observed seasonal patterns in stroke incidence and severity. However, the underlying mechanisms and the independent contributions of seasonal factors remain incompletely characterized.

Hemoglobin A1c (HbA1c) provides a more comprehensive reflection of glycemic control compared to point-in-time FPG measurements. Seasonal variations in HbA1c levels have been documented among patients with diabetes (Cheng et al. 2023; Raphael et al. 2021), with important clinical implications since each 1% absolute increase corresponds to an 18% elevation in cardiovascular event risk (Lattanzi et al. 2016). Notably, even transient seasonal HbA1c elevations may have cumulative vascular consequences. Evidence from diverse regions, including Japan(Sakamoto et al. 2019), Korea (Kim et al. 2014), China(Liang 2007), Portugal (Pereira et al. 2015), the United Kingdom(Carney et al. 2000), and the United States(Tseng et al. 2005), reveals consistent winter peaks in HbA1c levels exhibit a seasonal decline of 0.13% to 0.6%, particularly in climates with subzero winter temperatures. Despite these advances, few studies have systematically examined glucose parameter variations in acute ischemic stroke (AIS) populations. Furthermore, comprehensive analyses integrating meteorological data with glucose parameter and stroke severity are lacking.

Therefore, in the current study, we investigated seasonal and monthly variations in blood glucose levels among AIS patients across selected areas of the Tianjin district of China. Additionally, we evaluated the influence of meteorological parameters on these seasonal patterns and explored the potential associations with clinical outcomes. Our findings provide novel insights into environmental determinants of AIS risk and opportunities for seasonally-tailored prevention strategies.

## Materials and methods

### Study area and regional climate

Tianjin Municipality is located in the northeast North China Plain between 38°34′−40°15 N and 116°43′།118°194′ E. It is an important component of the Beijing-Tianjin-Hebei (BTH) city cluster with a total area of 11,947 km^2^ and a resident population of 15,568,700. The area belongs to the continental monsoon climate. The average annual temperature is between 10℃ and 12℃, with the mean temperature of − 1.9℃ in January and 26.4℃ in July. The weather in Tianjin follows 4 distinct seasons, winter, spring, summer, and autumn. Hexi District, one of Tianjin’s central urban districts, serves as the municipal administrative hub while functioning concurrently as the city’s premier financial and cultural center. Within this strategically vital district, the Second Hospital of Tianjin Medical University maintains exclusive designation as the Advanced Stroke Center.

### Meteorological data

The meteorological variables studied included daily mean ambient temperature (maximum and minimum) of the 24-hr calendar day period (0:00AM-11:59PM), monthly measures of mean temperature and diurnal temperature range (the difference between the monthly average maximum and minimum temperatures) for the 10-year study period, which were obtained from the Meteorological Administration of Tianjin. The division of seasons was made according to the local criteria, which was basically based on the temperature patterns. Spring was counted from 1 March to 31 May, summer from 1 June to 31 August, autumn from 1 September to 30 November and winter from 1 December to 28 February.

### Study population

**Fig. 1:**
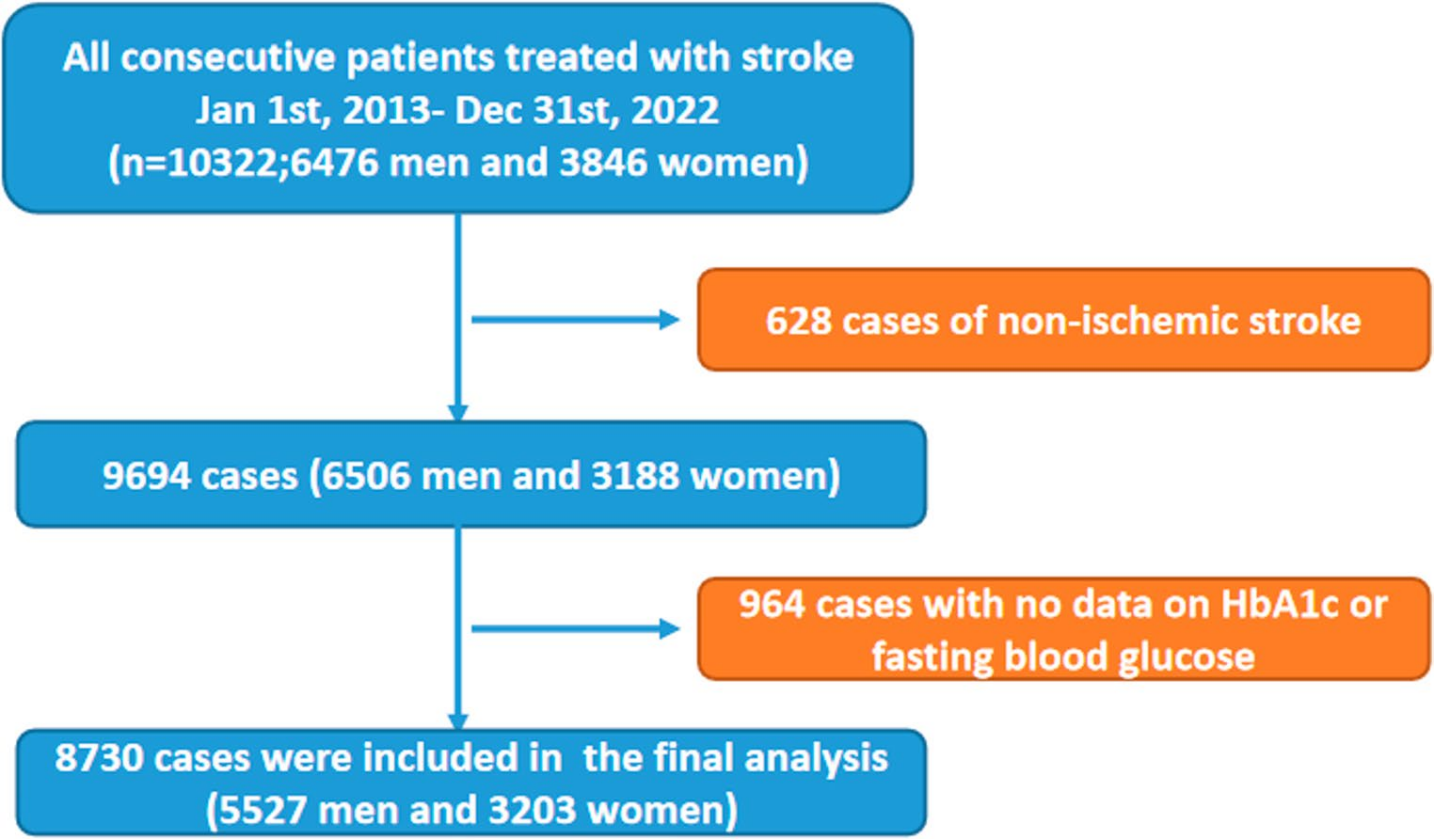
Flowchart of study enrollment

To ascertain the variation of blood glucose and HbA1c levels in different seasons, we retrospectively searched the records based on the Second Hospital of Tianjin Medical University from January 2013 to December 2022 and identified 9694 consecutive patients who were admitted with AIS. No exceptional events concerning weather or environment were noted in this period. We then excluded 964 patients who were not eligible for this study for the reasons of no blood samples were collected to detect HbA1c or FPG. A total of 8730 patients diagnosed as AIS were finally enrolled, of whom 5864 were non-diabetic patients and another 2866 were diabetic patients with cerebral infarction (Fig. 1). Stroke was defined according to the definition of the World Health Organization (WHO) Statistical analysis. Diagnosis was confirmed by CT or MRI in all cases.

### Data collection

Demographic and clinical data of age, sex, body mass index (BMI), medical history, blood pressure, results of laboratory tests and admission National Institute of Health Stroke Scale (NIHSS) score were collected. Diabetes mellitus was defined as HbA1c levels of above 6.5%, FPG levels of above 7 mmol/L, or the use of anti-diabetic medication. The first systolic and diastolic blood pressure (SBP, DBP) measured on admission was used for the analyses. Blood samples were collected from the antecubital vein in the morning after an overnight fasting period (> 8 h) and transfused into vacuum tubes containing Ethylene Diamine Tetraacetic Acid (EDTA). Physical activity was assessed as participation in regular sporting activities at least once a week for a minimum of 30 min. BMI was defined as body weight (kg) divided by the square of height (meters). Admission severity was measured using NIHSS scores. The NIHSS scores were classified into three categories and proportion of patients: A score from 0 to 3 was defined as the mild level, 4–7 as the moderate level and > 7 as the severe level. After enrollment, patients were divided into four groups based on the onset seasons: spring, summer, autumn and winter.

### Statistics analysis

We performed all statistical analyses using IBM SPSS Statistics for Macintosh, Version 26.0 (IBM Corp., Armonk, New York, USA) and R software (version 4.4.2). Continuous data were expressed as mean ± standard deviation (SD) or median and interquartile range (IQR). Categorical data were presented as absolute values and percentages. Chi-test was used to analyze distribution of frequencies, while one-way ANOVA was used for normally distributed continuous variables comparisons, followed by the Tukey–Kramer post hoc test. The Kruskal-Wallis H test was used for comparisons of non-normally distributed continuous variables between groups. The general linear model was applied for calculating the seasonal effects on glycemic parameters. We applied a 3-month lag to HbA1c measurements based on its established correlation with mean glucose over erythrocytes’ 90–120 day lifespan, with the peak correlation at 12 weeks, following international glycemic assessment guidelines (ElSayed et al. 2023).

A generalized additive model (GAM) was applied to assess the association between ambient temperature, glycemic parameters and stroke severity at admission, quantified by the NIHSS score. In this model, temperature (or FPG level, HbA1c level) was incorporated as a smooth term using natural cubic splines, adjusted for potential confounders. The model is formulated as:

1 $$\:\begin{array}{c}{NIHSS}_i={\beta\:}_0+ns\left(exposure,df\right)+\sum\:_k{\beta\:}_k{COVs}_{ki}+{\epsilon\:}_i\end{array}$$

Where $\:{NIHSS}_{i}$ denotes the NIHSS score for patients i; ns(⋅) represents the natural cubic spline function applied to temperature, FPG level or HbA1c level, with the degrees of freedom set between 3 and 5 based on clinical knowledge; $\:{COVs}_{ki}$indicates the set of covariates, including age, sex, season, relative humidity, atmospheric pressure, medical history, systolic blood pressure (SBP), diastolic blood pressure (DBP), total cholesterol (TC), triglycerides (TG), low-density lipoprotein cholesterol (LDL-C), high-density lipoprotein cholesterol (HDL-C), creatinine, uric acid (UA), body mass index (BMI), lipid lowering drugs which were known to influence stroke outcomes based on current evidence; and $\:{\epsilon\:}_{i}$ is the error term. All statistical tests were two-tailed, and *P* < 0.05 was considered statistically significant.

To investigate the potential interaction with season, we further employed a stratified model including an interaction term between exposure and season. All other covariates and parameter settings remained consistent with those in Eq. (1). The extended model is specified as:

2 $$\:\begin{array}{c}{NIHSS}_i={\beta\:}_0+ns\left(exposure,df\right):season+\sum\:_k{\beta\:}_k{COVs}_{ki}+{\epsilon\:}_i\end{array}$$

All statistical tests were two-tailed, and *P* < 0.05 was considered statistically significant.

## Results

### Characteristics of study subjects and seasonal patterns

**Fig. 2:**
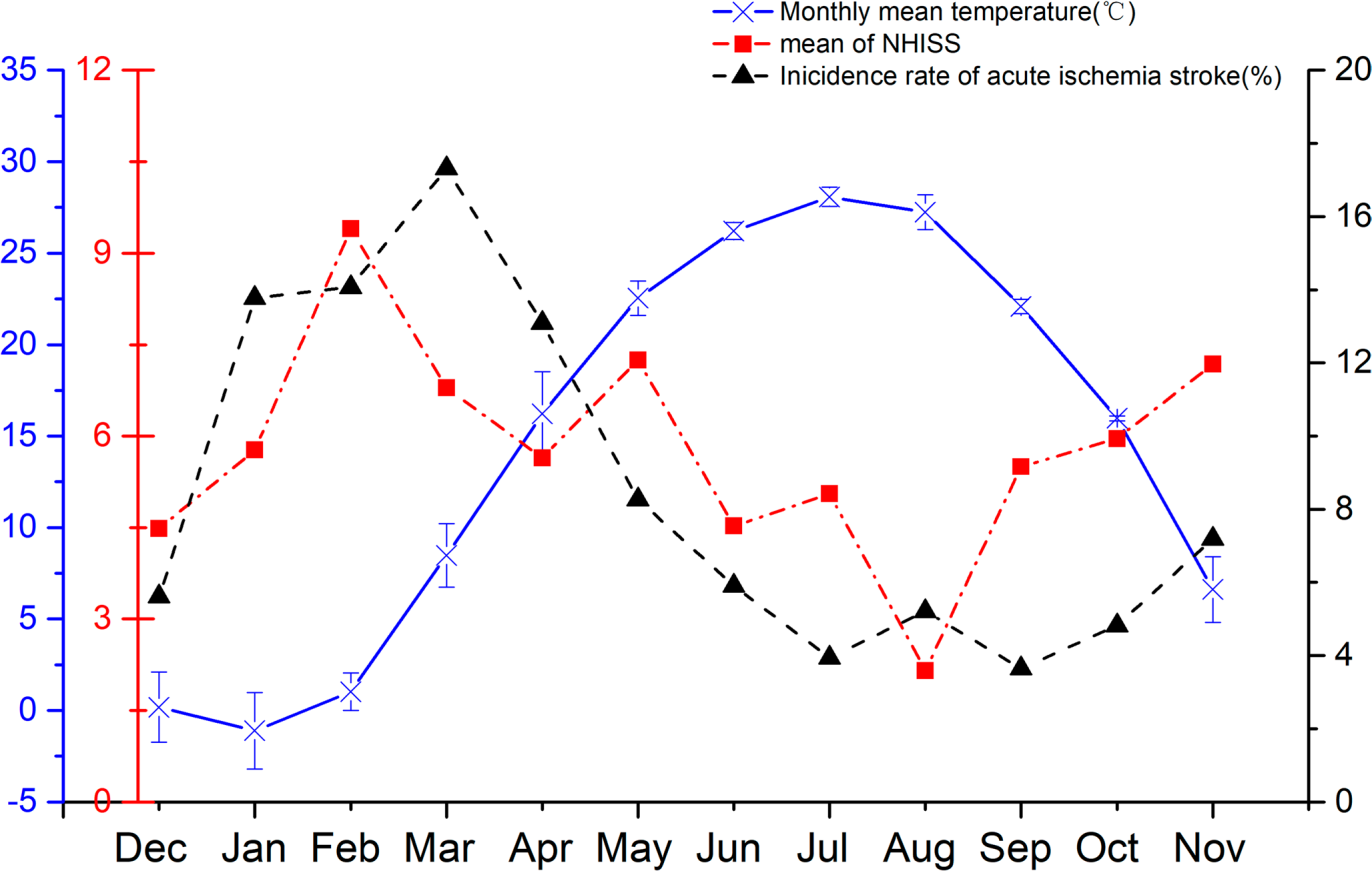
Temperature, NIHSS scores, and AIS incidence across months

Table 1 provided baseline characteristics of the participants. Over the 10-year study period, 8730 AIS patients occurred in 5527 male patients, wherein 62.20% were ≥ 65 years old. Significant seasonal variations were observed in several clinical parameters, including body mass index (BMI, *P* = 0.013), systolic blood pressure (SBP, *P* = 0.027), diastolic blood pressure (DBP, *P* = 0.045), and FPG levels in diabetic patients (*P* = 0.033). Analysis of clinical outcomes revealed distinct seasonal patterns, with poorer outcomes predominantly observed during spring and winter compared to summer and autumn. Among diabetic patients, the winter group exhibited the highest median NIHSS score of 7, while the summer group showed the lowest median score of 5. The proportion of patients admitted with NIHSS scores > 7 varied seasonally: 31.8% in winter, 18.8% in spring, 14.7% in summer, and 20.7% in autumn. Figure 2 showed monthly variations in mean temperature, NIHSS scores and AIS incidence. From November to March, lower temperatures coincide with increased AIS incidence and NIHSS scores, suggesting links between temperature, stroke occurrence and severity.

**Table 1:** Baseline characteristics of study participants distributed by season

	Total(*n* = 8730)	Spring(*n* = 2226)	Summer(*n* = 1641)	Autumn(*n* = 2169)	Winter(*n* = 2694)	*P* value
Days per year	365	92	92	91	90	-
First-ever stroke cases (%)	6277(71.90)	1606(72.15)	1128(68.74)	1560(71.91)	1983(73.61)	0.813
Male (%)	5527(63.31)	1414(63.51)	1069(65.17)	1370(63.16)	1674(62.15)	0.626
Age, years	70.61 ± 12.07	71.75 ± 11.42	69.04 ± 12.24	69.44 ± 12.76	71.04 ± 12.28	0.875
Age, years(%)						
<65	3298(37.78)	873(39.23)	630(38.42)	832(38.38)	963(35.76)	0.208
65–80	3658(41.90)	865(38.85)	725(44.21)	920(42.42)	1148(42.61)	
≥ 80	1772(20.30)	488(21.92)	285(17.37)	416(19.20)	583(21.63)	
Body mass index (kg/m2)	25.58 ± 3.49	25.8 ± 3.21	24.3 ± 3.30^a^	25.1 ± 3.92	26.6 ± 3.57^b^	**0.013**
Blood pressure (mmHg)						
Systolic	151.98 ± 21.69	153.69 ± 22.39	146.14 ± 20.92^a^	151.26 ± 22.48 ^b^	154.73 ± 23.06^b^	**0.027**
Diastolic	85.10 ± 12.91	86.79 ± 13.67	81.92 ± 12.84^a^	85.48 ± 13.25^b^	85.38 ± 12.91^b^	**0.045**
FPG (mmol/L)						
Diabetes	9.75(9.39, 10.1)	9.46(8.60, 10.32)	9.07(8.45, 9.70)^a^	9.50(8.56, 10.44)^b^	10.29(9.74, 10.84)^abc^	**0.033**
Non-diabetes	5.20(5.14, 5.25)	5.14(5.00, 5.28)	5.27(5.16, 5.38)	5.19(5.06, 5.32)	5.17(5.05, 5.28)	0.339
HbA1c						
Diabetes	8.04(7.89, 8.18)	8.07(7.75, 8.39)	8.02(7.68, 8.35)	8.01(7.53, 8.48)	8.05(7.83, 8.27)	0.764
Non-diabetes	5.70(5.67, 5.72)	5.69(5.63, 5.75)	5.75(5.69, 5.80)	5.66(5.59, 5.71)	5.68(5.63, 5.74)	0.239
Diabetes (%)	5615(64.32)	1438(64.62)	1071(65.26)	1358(62.63)	1748(64.88)	0.877
Hyperlipemia(%)	3463(39.67)	869(39.05)	660(40.20)	861(39.68)	1073(39.84)	0.488
Chronic kidney disease (%)	1527(17.49)	391(17.57)	276(16.83)	374(17.26)	486(18.04)	0.579
Physical activity(%)	1113(12.75)	327(14.69)	203(12.39)	293(13.52)	290(10.78)	0.179
Current smoking (%)	2510(28.75)	582(26.13)	462(28.15)	633(29.18)	833(30.93)	0.432
Current alcohol drinking (%)	1329(15.22)	316(14.21)	226(13.78)	340(15.69)	447(16.59)	0.544
Anti-diabetic drugs (%)						
Insulin	1238(14.18)	327(14.71)	236(14.36)	304(14.02)	371(13.78)	0.713
Oral drugs only	2755(31.56)	708(31.82)	508(30.95)	674(31.07)	865(32.12)	0.625
Anti-hypertensive drugs (%)	4936(56.54)	1266(56.89)	939(57.20)	1198(55.24)	1533(56.90)	0.335
Lipid-lowering agents (%)	1396(15.99)	358(16.07)	253(15.43)	346(15.96)	439(16.28)	0.417
NIHSS score						
Diabetes	6(2, 10)	6(1, 10)	5(2, 9)	5(2, 10)	7(2, 10)^abc^	**0.021**
Non-diabetes	4(1, 9)	5(2, 9)	3(1, 8)	4(2, 8)	6(2, 10)	0.065
Severity levels (%)						
NIHSS 0–3	3953(45.28)	1139(51.16)	829(50.51)	1014(46.75)	944(35.05%)	**<0.001**
NIHSS 4–7	2892(33.13)	673(30.23)	580(35.34)	745(34.36)	894(33.17%)	
NIHSS >7	1574(18.03)	419 (18.84)	241(14.70)	49(20.68)	865(31.78)	
Clinical outcomes						
Death (%)	502(5.75)	122(5.46)	75(4.56)	112(5.18)	193(7.17)	0.062

Data is mean ± standard deviation, number (percentage) or median (interquartile range)

^a^ *P* < 0.05 vs. spring; ^b^ *P* < 0.05 vs. summer; ^c^ *P* < 0.05 vs. autumn. FPG, fasting plasma glucose; NIHSS, National Institute of Health Stroke Scale. Bold values indicate statistical significance

Table 2 summarized the meteorological and glucose parameters during the study period, including FPG, HbA1c, daily mean temperature, air pressure, relative humidity, and wind speed. Diabetic patients demonstrated significantly higher serum levels of FPG and HbA1c compared to non-diabetic individuals. The mean environmental conditions were as follows: temperature 15.05 °C, air pressure 1016.17 hPa, relative humidity 51.73%, and wind speed 1.73 m/s. Table 3 provided a comprehensive overview of temperature variations across different time scales.

**Table 2:** Summary statistics of glucose levels and weather conditions during 2013–2022

Variables	Minimum	5%	25%	50%	75%	95%	Maximum	Mean	SD	*P* values for difference of means
FPG(mmol/L)										
All	1.15	4.18	4.96	5.84	7.72	14.46	34.33	7.02	3.55	-
Diabetes	1.93	4.58	5.95	7.51	9.64	15.17	29.80	8.35	3.43	<0.001
Non-diabetes	1.15	4.13	4.65	5.12	5.67	6.60	6.99	5.18	0.78	
HbA1c(%)										
All	4.30	5.20	5.60	6.10	7.20	10.40	14.30	6.67	1.63	-
Diabetes	4.60	5.40	6.30	7.10	8.40	11.30	14.30	7.55	1.79	<0.001
Non-diabetes	4.30	5.10	5.40	5.70	5.90	6.30	6.60	5.68	0.36	
Temeparature(℃)	−13.85	−2.17	5.23	15.32	25.24	29.49	33.53	15.05	10.83	-
Air pressure(hPa)	994.05	1000.98	1006.96	1016.61	1024.23	1033.59	1043.35	1016.17	10.38	-
Relative humidity(%)	12.00	21.85	37.30	51.63	66.32	81.56	94.21	51.73	18.45	-
Wind speed(m/s)	0.56	0.87	1.24	1.56	2.03	3.10	7.65	1.73	0.76	-

**Table 3:** Summary statistics of ambient temperature in months and seasons during 2013–2022

Time scale	Mean	SD	Minimum	Maximum
Month				
January	−1.61	2.17	−5.40	4.30
February	−0.92	3.64	−6.20	5.40
March	8.48	1.81	5.60	12.60
April	16.31	5.54	7.07	23.68
May	22.13	3.06	17.80	28.54
June	26.50	3.71	19.02	32.50
July	28.81	2.22	25.59	33.35
August	28.27	2.15	24.66	33.43
September	23.12	3.40	16.69	28.33
October	14.97	3.02	10.47	20.53
November	7.98	1.96	4.66	11.59
December	−1.24	4.65	−7.49	8.15
Season				
Spring	17.20	6.54	5.60	28.54
Summer	27.87	2.90	19.02	33.43
Autumn	15.80	6.78	4.66	28.33
Winter	−1.29	3.51	−7.49	8.15

## Seasonal variations in the level of FPG

**Fig. 3:**
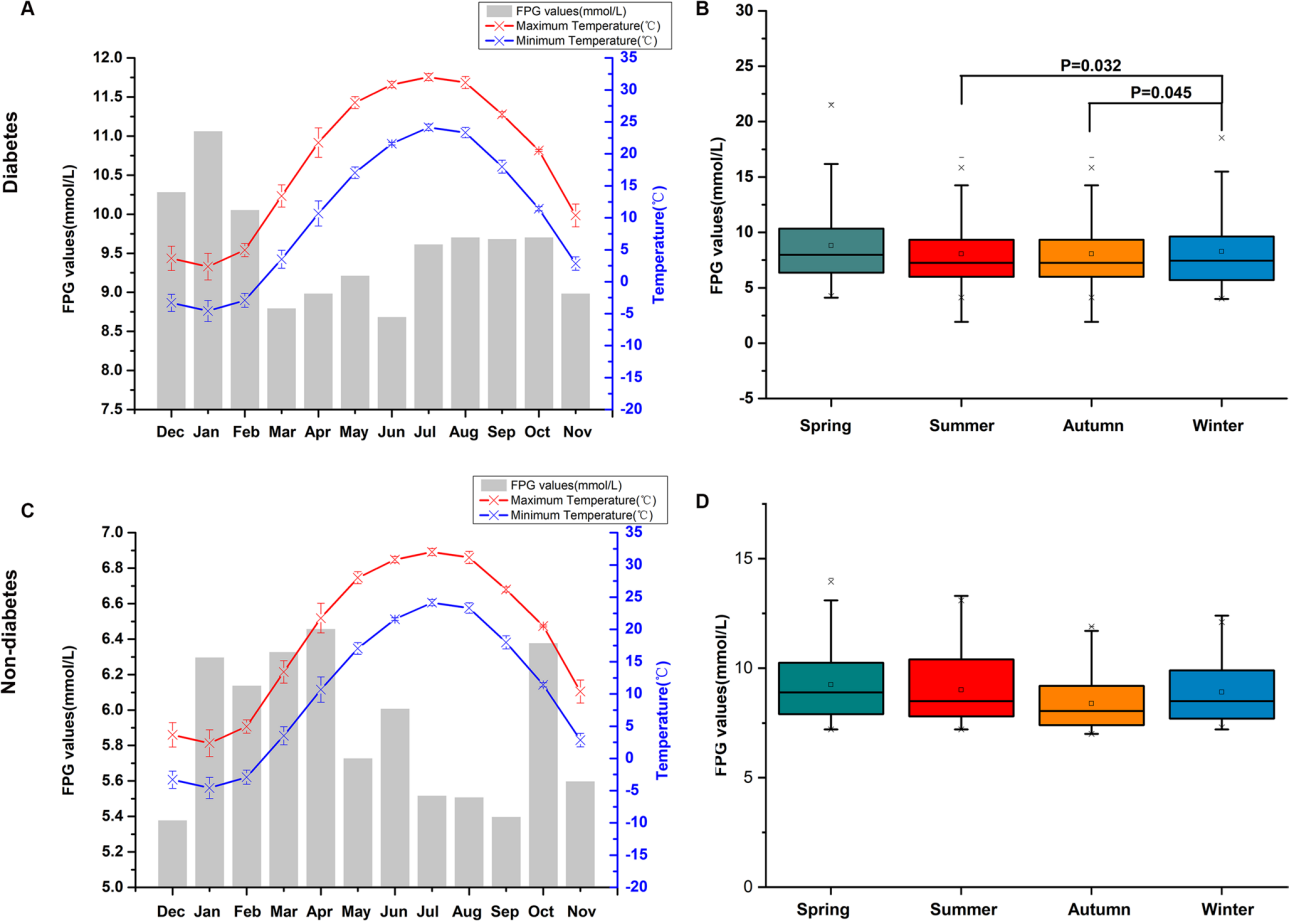
Monthly mean FPG levels and seasonal changes (**a**) Monthly mean FPG levels in patients with diabetes; (**b**) Seasonal variation in FPG levels in patients with diabetes; (**c**) Monthly mean FPG levels in patients without diabetes; (**d**) Seasonal variation in FPG levels in patients without diabetes

Figure 3 illustrated the monthly variations in FPG levels in relation to ambient temperature. Additionally, we analyzed FPG levels using box-plot diagrams with descriptive statistics, stratified by quartile groups of meteorological factors. The analysis revealed significant seasonal fluctuations in FPG levels across the study population. Specifically, FPG levels exhibited an inverse relationship with temperature, demonstrating lower values during warmer months and higher values during colder months. Among diabetic patients, the peak FPG level was observed in January (11.17 mmol/L), while the lowest level was recorded in June (8.69 mmol/L). FPG levels were significantly higher in winter compared to summer (*P* = 0.032) and autumn (*P* = 0.045). A statistically significant increase in FPG levels was observed during winter compared to summer, with a mean difference of 1.22 mmol/L (95% *CI*: 1.10–1.29 mmol/L; *P* = 0.005).

### Seasonal variations in the level of HbA1c and its consistent cyclic variation with FPG

**Fig. 4:**
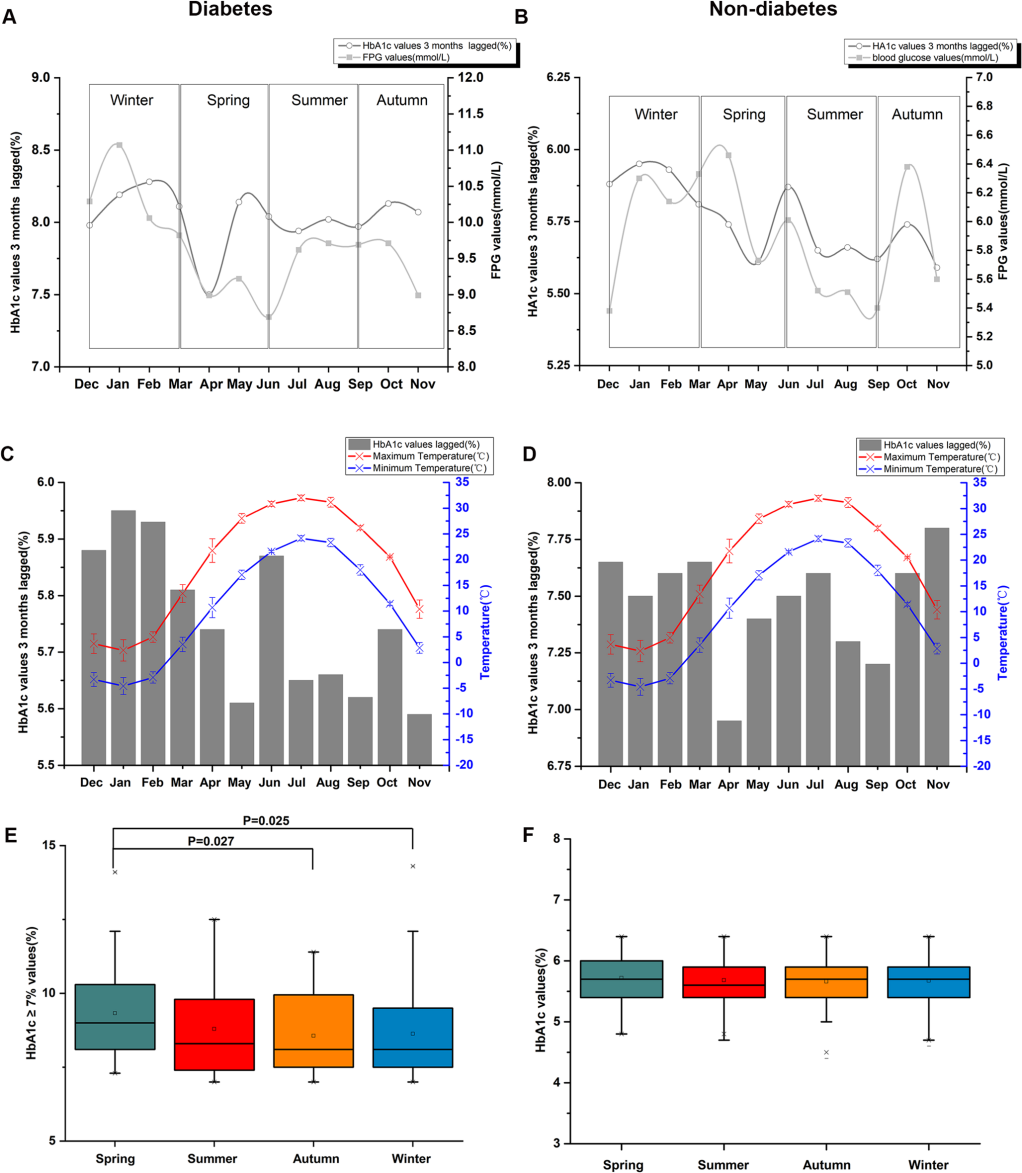
Monthly mean HbA1c values and seasonal changes (**a**) Linear trend between FPG levels and HbA1c values 3 months lagged in patients with diabetes; (**b**) Linear trend between FPG levels and HbA1c values 3 months lagged in patients without diabetes; (**c**) Monthly mean HbA1c values 3 months lagged in patients with diabetes; (**d**) Monthly mean HbA1c values 3 months lagged in patients without diabetes; (**e**) Seasonal variation in HbA1c values greater than 7.0% in patients with diabetes; (**f**) Seasonal variation in HbA1c values in patients without diabetes

Furthermore, we applied a 3-month lag to the monthly average HbA1c values. These lagged variables were incorporated into a linear trend model to assess the correlation between HbA1c and FPG. As illustrated in Fig. 4A and B, HbA1c values exhibited significant monthly fluctuations that were closely associated with FPG levels. After adjusting for linear trends, the current average HbA1c values were found to be significantly influenced by FPG levels from 3 months prior. Notably, early spring exhibited a significant increase in HbA1c levels, while late summer showed a marked decrease (Fig. 4C and D). The analysis revealed significantly higher HbA1c levels during spring compared to autumn and winter in diabetes patients with suboptimal glycemic control (HbA1c > 7.0%), as shown in Fig. 4E. Seasonal fluctuation quantitative assessment demonstrated that diabetic patients had significantly elevated HbA1c levels during spring, showing 0.77 units (95% *CI*: 0.59–0.92; *P* = 0.027) and 0.81 units (95% *CI*: 0.61–1.02; *P* = 0.025) increases compared to winter and autumn measurements, respectively. However, no significant seasonal fluctuations were observed among patients who achieved the goal of HbA1c < 7%. Given that HbA1c reflects average blood glucose levels over approximately 3 months, these findings suggested that both extreme cold temperatures and large diurnal temperature variations may contribute to increased blood glucose levels, potentially explaining the observed seasonal patterns in glucose control.

### The effects of seasons on FPG and HbA1c

After adjustment for age, sex, BMI, and other covariates, the summer group exhibited 0.083 units lower FPG levels (95% *CI*: −0.726, − 0.062; *P* = 0.032) and the autumn group showed 0.125 units lower FPG levels (95% *CI*: −0.590, − 0.073; *P* = 0.012) compared to the winter group (Table 4). Meanwhile, compared to the autumn group, the spring group had 0.201 units higher HbA1c levels (95% *CI*: 0.099, 0.557; *P* < 0.001), and the summer group had 0.107 units higher HbA1c levels (95% *CI*: 0.096, 0.449; *P* = 0.032) (Table 5).

**Table 4:** Estimates of season effects on FPG levels in the general linear model

Variable	Model 1		Model 2	
	β coefficien (95% CI)	*P* value	β coefficient (95% CI)	*P* value
Spring	−0.071(−1.451,0.358)	0.236	−0.023(−0.944, 0.583)	0.583
Summer	−0.124(−0.971,−0.033)	0.036	−0.083(−0.726,−0.062)	0.042
Autumu	−0.176(−0.778,−0.160)	0.003	−0.125(−0.590,−0.073)	0.012
Winter(reference)	0		0	
Covariates				
Age	-		0.042(0.034,0.058)	0.021
Male	-		0.374(−0.081,0.829)	0.107
BMI	-		0.115(0.087,0.149)	0.017
Physical activity	-		−0.345(−0.627,−0.063)	0.041
Current smoking	-		−0.013(−0.029,0.003)	0.117
SBP	-		0.0142(−0.022.0.050)	0.445
DBP	-		0.087(−0.084,0.257)	0.318
Combined with diabetes	-		1.406(0.932,1.480)	<0.001
Using anti-diabetic drugs	-		−0.806(−1.035,−0.602)	<0.001
Serum creatinine	-		0.078(0.036,0.120)	0.030
Low density lipoprotein	-		−0.010(−0.034,0.012)	0.335
NIHSS scores	-		0.041(0.011,0.089)	0.034

Model 1 was unadjusted; Model 2 was adjusted for age, sex, BMI, physical activity, current smoking, blood pressure, combined with diabetes, using anti-diabetic drugs, serum creatinine, low density lipoprotein and NIHSS scores. CI, confidential interval

**Table 5:** Estimates of season effects on HbA1c levels ≥ 7% in the general linear model

Variable	Model 1		Model 2	
	β coefficien (95% CI)	*P* value	β coefficient (95% CI)	*P* value
Spring	0.232(0.012, 0.447)	<0.001	0.201(0.099, 0.557)	<0.001
Summer	0.182(0.101, 0.492)	0.003	0.107(0.096, 0.449)	0.032
Autumu(reference)	0		0	
Winter	0.054(−0.077, 0.082)	0.112	0.017(−0.051, 0.049)	0.153
Covariates				
Age	-		0.202(0.035,0.368)	0.018
Male	-		−0.206(−0.592,0.179)	0.293
BMI	-		0.150(0.005.0.294)	0.042
physical activity	-		−0.027(−0.044,−0.011)	0.001
current smoking	-		−0.008(−0.023,0.006)	0.117
SBP	-		−0.008(−0.023,0.006)	0.241
DBP	-		0.005(−0.026,0.339)	0.759
combined with diabetes	-		0.785(0.531,1.139)	<0.001
using anti-diabetic drugs	-		−0.503(−0.695,−0.321)	<0.001
serum creatinine	-		0.007(0.004,0.009)	<0.001
low density lipoprotein	-		−0.002(−0.020,0.016)	0.842
NIHSS scores	-		0.037(0.001,0.073)	0.044

Model 1 was unadjusted; Model 2 was adjusted for age, sex, BMI, physical activity, current smoking, blood pressure, combined with diabetes, using anti-diabetic drugs, serum creatinine, low density lipoprotein and NIHSS scores. CI, confidential interval

### Association between glycemic parameters and seasonal variations in admission stroke severity

**Fig. 5:**
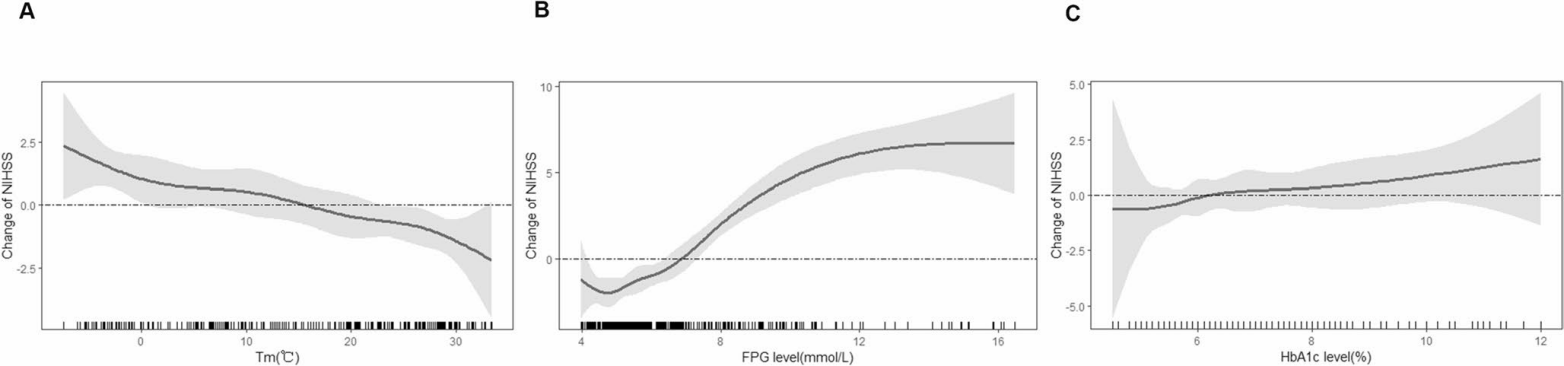
Association between glycemic parameters and seasonal variations in admission stroke severity (**a**) Curve fitting plot between daily average temperature and NIHSS scores. The Gaussian generalized additive model is adjusted according to age, gender, season, relative humidity, atmospheric pressure and past history; (**b**) Curve fitting plot between FBG and NIHSS scores. The Gaussian generalized additive model is adjusted according to age, gender, season, past history, SBP, DBP, TC, TG, LDL-C, HDL-C, Creatinine, UA, HbA1c, BMI, lipid lowering drugs, antidiabetic drugs, and antiplatelet drugs; (**c**) Curve fitting plot between HbA1c and NIHSS scores. The Gaussian generalized additive model is adjusted according to age, gender, season, past history, SBP, DBP, TC, TG, LDL-C, HDL-C, Creatinine, UA, FPG, BMI, lipid lowering drugs, antidiabetic drugs, and antiplatelet drugs. The shadow represents the 95% CI confidence interval

Figure 5A demonstrated the exposure response curves between mean temperatures and admission NIHSS scores, which seems to be non-linear negative correlation. It means that as the temperature decreased, the admission NIHSS scores increased. Of note, 1 ◦C decrease of mean temperature was associated with 2.5 (95% *CI*: −4.5, −0.5, *P* < 0.01) increase of admission NIHSS scores. After adjusting for age, sex, season, past history, SBP, DBP, TC, TG, LDL-C, HDL-C, Creatinine, UA, HbA1c, BMI, lipid lowering drugs, antidiabetic drugs, and antiplatelet drugs, the smooth curve fitting plot demonstrated a non-linear positive relationship between FPG and admission NIHSS scores (Fig. 5B). Specifically, higher FPG levels were positively correlated with increased stroke severity at admission, with each 0.1 mmol/L increment in FPG corresponding to a 0.8 rise in NIHSS scores (95% *CI*: 0.5, 1.1, *P* < 0.01). However, HbA1c values has no effect on admission NIHSS scores.

Table 6 showed interaction effects of temperature, FPG, HbA1c, and season as a categorical variable on NIHSS changes. Mean temperature interacted minimally with season, indicating consistent temperature effects across seasons. By contrast, glycemic parameters showed seasonal variation: FPG associated more strongly with NIHSS in winter versus summer and autumn, while HbA1c effects differed significantly between autumn and winter. These findings highlighted the need to consider seasonal modulation of glycemic impacts on stroke severity.

**Table 6:** Interaction effects between temperature, glycemic parameters and season by stratification analysis

Predictor	Comparisons					
	Spring-summer	Spring-autumn	Spring-winter	Summer-autumn	Summer-winter	Autumn-winter
Temperature	−0.043(−0.926,0.840)	0.688(−0.335,1.710)	2.518(−1.272.6.308)	0.730(−0.112,1.573)	2.561(−1.022,6.144)	1.831(−1.874,5.535)
FPG	0.450(−0.628,1.530)	0.804(−0.246,1.855)	−1.165(−2.227, −0.103)	0.353(−0.666,1.373)	**−1.616(−2.648-0.583)**	**−1.969(−3.003**,**−0.935)**
HbA1c	0.596(−0.625,1.817)	0.926(−0.325,2.177)	−0.805(−2.012,0.402)	0.330(−0.864,1.524)	−1.400(−2.553,−0.247)	**−1.731(−2.913**,**−0.549)**

## Discussion

In this population-based stroke registry study, we identified significant seasonal variations in both FPG and HbA1c levels among AIS patients, particularly those with diabetes. The results demonstrated that mean FPG concentrations exhibited a distinct seasonal pattern, peaking in January (11.07 mmol/L) and reaching their lowest levels in June (8.69 mmol/L). Notably, AIS patients with diabetic displayed more pronounced fluctuations, with winter FPG levels being approximately 1.22 mmol/L higher than those in summer. FPG levels were markedly lower in both summer (*β* = −0.083, 95% *CI*: −0.726, −0.062, *P* = 0.042) and autumn (*β* = −0.125, 95% *CI*: −0.590, −0.073, *P* = 0.012) when compared to winter. Our findings were consistent with previous studies demonstrating seasonal variations in glycemic control among diabetic patients, characterized by higher glucose levels in winter and lower levels in summer (Belsare et al. 2023; Takai et al. 2020). This observation aligned with a study of 49,417 participants, which revealed a U-shaped relationship between ambient temperature and FPG levels, with particularly elevated glucose concentrations during winter months (Li et al. 2016). A multi-center Chinese study analyzing 1.4 million physical examination population confirmed both the seasonal discrepancy in FPG levels and a distinct north-south gradient, with northern regions exhibiting higher winter glucose values than southern areas (Zhang et al. 2021).

Meanwhile, a comparable seasonal trend was observed for HbA1c levels, especially in patients with suboptimal glycemic control (HbA1c > 7.0%). The difference between the highest HbA1c value (observed in spring) and the lowest (observed in autumn) was approximately 0.81 units. HbA1c exhibited a significant upward trend in early spring and a downward trend in late summer, underscoring the dynamic nature of glycemic control across seasons. HbA1c levels were significantly higher in spring (*β* = 0.201, 95% *CI*: 0.099, 0.557, *P* < 0.001) and summer (*β* = 0.107, 95% *CI*: 0.096, 0.449, *P* = 0.032) compared to autumn. The spring HbA1c peak likely reflected the delayed integration of winter hyperglycemia (December–February), when cold temperatures exacerbated glycemic dysregulation. Conversely, improved glucose control during warmer months manifests as lower autumn HbA1c. To our knowledge, this represented the first investigation of temporal associations between HbA1c and FPG within the context of ambient temperature variations, while confirming the established 2–3 month glycemic monitoring role of HbA1c (Ahuja et al. 2024).

The above results indicated that although the extreme temperature difference might affect the fluctuation of blood glucose, the overall mean blood glucose levels remained highest in winter, which may further influence the incidence and severity of stroke. Higgin et al. (Higgins et al. 2009) found significant seasonal fluctuations in both northern and southern hemispheres (higher HbA1c in cold months and lower in warm months), while Singapore’s stable climate showed no fluctuations, confirming temperature’s influence on glycemic control. The biological mechanisms that underlay the seasonality in blood glucose levels might include seasonal variation in HbA1c and physiological changes. Studies have found that a complex of potential factors including the stressful to carbohydrate tolerance in winter, alteration in diet, decreased physical activity, exposure to sunlight, weight gain and increase in counter-insulin hormones (Iwata et al. 2024; Nicolo and Boullata 2019; Banihani et al. 2020), could partially explain the seasonal variation of blood glucose levels. These findings highlighted the importance of considering seasonal and environmental factors in the management of diabetes and stroke prevention strategies.

Furthermore, our findings corroborated a significant nonlinear negative correlation between ambient temperature and NIHSS scores (95% CI: −4.5,−0.5; *P* < 0.01), while establishing a positive association between FBG levels and NIHSS scores (95% CI: 0.5, 1.1, *P* < 0.01). A growing body of evidence had identified an association between elevated glucose at admission and increased mortality or poor outcome following AIS (Kim et al. 2023; Shi et al. 2025; Zhang et al. 2024). Post-stroke hyperglycemia was known to correlate with stroke severity, potentially mediated by a stress-induced cortisol response (Mosenzon et al. 2023). Hyperglycemia may exacerbate ischemic brain injury by amplifying inflammatory responses, thereby promoting neuronal damage(Climent et al. 2024; Bains et al. 2023). At the vascular level, dysregulation of glucose metabolism induced endothelial dysfunction through oxidative stress-mediated pathways, accelerating atherogenesis and increasing vulnerability to acute cerebrovascular incidents(Karakasis et al. 2025). The present study showed notable seasonal variations in admission severity and clinical outcomes, with more severe neurological deficits observed in winter. This temporal pattern aligned with the hypothesis that acute hyperglycemia, as reflected by elevated FPG levels, may exacerbate ischemic injury and contribute more directly to early neurological impairment. In contrast, no statistically significant relationship was observed between HbA1c levels and admission NIHSS scores. The lack of association between current HbA1c levels and stroke severity might reflect the temporal mismatch between this chronic glycemic marker (reflecting 2–3 month exposure) and acute neurological outcomes. However, we cannot exclude the possibility that longer-term glycemic control may influence baseline stroke vulnerability through cumulative cerebrovascular effects. The robust association between FPG and outcomes highlighted the need for further investigation into targeted glycemic control strategies during the acute phase of ischemic stroke.

Despite its significant insights, our single hospital-based study was not without limitations. Firstly, as a retrospective analysis, potential biases in data collection and residual confounding factors might exist. Secondly, selection bias might have occurred since cases who died before hospitalization or sought treatment elsewhere were excluded. Thirdly, unmeasured lifestyle factors (e.g., dietary patterns and physical activity) could potentially confound the observed seasonal glucose variations. Most importantly, the establishment of causality required prospective cohort studies with larger sample sizes due to the cross-sectional nature of our research. While acknowledging these limitations, the rigorous exclusion of potential confounding factors and clinical validation strengthened the reliability of our findings. Future investigations should incorporate longitudinal designs to establish causal relationships between glycemic parameters and AIS progression. Furthermore, molecular-level studies were warranted to elucidate the precise mechanisms, particularly through characterization of involved inflammatory pathways. Such mechanistic insights might reveal novel therapeutic targets for glycemic control in stroke management.

## Conclusions

In conclusion, this 10-year longitudinal study demonstrated a consistent cyclic pattern in FPG and HbA1c levels, with significant seasonal and monthly variations. Notably, extreme cold temperatures and large diurnal temperature differences were associated with elevated blood glucose, which in turn correlated with increased stroke incidence and severity. These findings highlight the potential influence of climatic factors on metabolic dysregulation and cerebrovascular risk. Further research is warranted to elucidate the underlying pathophysiological mechanisms and explore their implications for the management of AIS, particularly in high-risk populations exposed to extreme weather conditions.

## Supplementary Information

Below is the link to the electronic supplementary material.

Supplementary Material 1 (PDF 1.65 MB)

## Data Availability

The data that support the findings of this study are available from the corresponding author upon reasonable request.

## References

[CR1] Ahuja S, Sugandha S, Kumar R, Zaheer S, Singh M (2024) Seasonal variation of HbA1c levels in diabetic and non-diabetic patients. Pract Lab Med 40:e00396. 10.1016/j.plabm.2024.e00396 38711868 10.1016/j.plabm.2024.e00396 PMC11070616

[CR2] Bains NK, Huang W, French BR, Siddiq F, Gomez CR, Qureshi AI (2023) Hyperglycemic control in acute ischemic stroke patients undergoing endovascular treatment: post hoc analysis of the stroke hyperglycemia insulin network effort trial. J Neurointerv Surg 15(4):370–374. 10.1136/neurintsurg-2021-018485 35414602 10.1136/neurintsurg-2021-018485

[CR3] Banihani SA, Fashtaky RA, Makahleh SM, El-Akawi ZJ, Khabour OF, Saadeh NA (2020) Effect of fresh pomegranate juice on the level of melatonin, insulin, and fasting serum glucose in healthy individuals and people with impaired fasting glucose. Food Sci Nutr 8(1):567–574. 10.1002/fsn3.1344 31993180 10.1002/fsn3.1344 PMC6977483

[CR4] Belsare P, Bartolome A, Stanger C, Prioleau T (2023) Understanding temporal changes and seasonal variations in glycemic trends using wearable data. Sci Adv 9(38):eadg2132. 10.1126/sciadv.adg2132 37738344 10.1126/sciadv.adg2132 PMC10516495

[CR5] Bian K, Hou C, Jin H, Feng X, Peng M, Zhao X, Yuan X, Sun W, Feng H, Xu G (2025) Association between triglyceride-glucose indices and ischemic stroke risk across different glucose metabolism statuses. Diabetes Res Clin Pract 222:112064. 10.1016/j.diabres.2025.112064 40010673 10.1016/j.diabres.2025.112064

[CR6] Carney TA, Guy SP, Helliwell CD (2000) Seasonal variation in HbA1c in patients with type 2 diabetes mellitus. Diabet Med 17(7):554–555. 10.1046/j.1464-5491.2000.00311-5.x 10972592 10.1046/j.1464-5491.2000.00311-5.x

[CR7] Cheng YC, Li YH, Liu HC, Hsu CY, Chang WJ, Lee IT, Lu CL (2023) The impact of a lockdown for the COVID-19 pandemic on seasonal HbA1c variation in patients with type 2 diabetes. Life. 10.3390/life13030763 10.3390/life13030763 PMC10054572 36983918

[CR8] Climent E, Rodriguez-Campello A, Jimenez-Balado J, Fernandez-Miro M, Jimenez-Conde J, Llaurado G, Ois A, Flores JA, Cuadrado-Godia E, Steinhauer EG, Chillaron JJ, Neurovascular Research G (2024) Acute-to-chronic glycemic ratio as an outcome predictor in ischemic stroke in patients with and without diabetes mellitus. Cardiovasc Diabetol 23(1):206. 10.1186/s12933-024-02260-9 38890732 10.1186/s12933-024-02260-9 PMC11186093

[CR9] ElSayed NA, Aleppo G, Aroda VR, Bannuru RR, Brown FM, Bruemmer D, Collins BS, Hilliard ME, Isaacs D, Johnson EL, Kahan S, Khunti K, Leon J, Lyons SK, Perry ML, Prahalad P, Pratley RE, Seley JJ, Stanton RC, Gabbay RA, American Diabetes A (2023) 6. Glycemic targets: standards of care in diabetes-2023. Diabetes Care 46(Suppl 1):S97–S110. 10.2337/dc23-S006 36507646 10.2337/dc23-S006 PMC9810469

[CR10] Fujii T, Arima H, Takashima N, Kita Y, Miyamatsu N, Tanaka-Mizuno S, Shitara S, Urushitani M, Miura K, Nozaki K (2021) Seasonal variation in incidence of stroke in a general population of 1.4 million Japanese: the Shiga Stroke Registry. Cerebrovasc Dis (1):7. 10.1159/000518370 10.1159/000518370 34515076

[CR11] He MZ, Kloog I, Just AC, Gutierrez-Avila I, Colicino E, Tellez-Rojo MM, Luisa Pizano-Zarate M, Tamayo-Ortiz M, Cantoral A, Soria-Contreras DC, Baccarelli AA, Wright RO, Yitshak-Sade M (2022) Intermediate- and long-term associations between air pollution and ambient temperature and glycated hemoglobin levels in women of child bearing age. Environ Int 165:107298. 10.1016/j.envint.2022.107298 35597113 10.1016/j.envint.2022.107298 PMC9233109

[CR12] Higgins T, Saw S, Sikaris K, Wiley CL, Cembrowski GC, Lyon AW, Khajuria A, Tran D (2009) Seasonal variation in hemoglobin A1c: is it the same in both hemispheres? J Diabetes Sci Technol 3(4):668–671. 10.1177/193229680900300408 20144310 10.1177/193229680900300408 PMC2769947

[CR13] Honda H, Igaki M, Komatsu M, Tanaka S-i (2021) Association between physical activity and seasonal variations in metabolic and vascular function in adults. Endocrines 2(2):150–159

[CR14] Huta-Osiecka A, Wochna K, Kasprzak Z, Nowak A (2021) Seasonal variation of 25-Hydroxyvitamin D and indices of carbohydrate and lipid metabolism in postmenopausal women. PeerJ 9:e11341. 10.7717/peerj.11341 34035990 10.7717/peerj.11341 PMC8126260

[CR15] Iwata S, Ashida K, Demiya M, Nagayama A, Hasuzawa N, Yoshinobu S, Sonezaki A, Yasuda J, Motomura S, Katsuki Y, Sugi K, Nomura M (2024) Preserved seasonal variation in glycemic control in patients with type 2 diabetes mellitus during COVID-19: a 3-year-long retrospective cohort study in older adults in Japan. BMC Endocr Disord 24(1):70. 10.1186/s12902-024-01602-8 38755559 10.1186/s12902-024-01602-8 PMC11100128

[CR16] Karakasis P, Theofilis P, Patoulias D, Vlachakis PK, Antoniadis AP, Fragakis N (2025) Diabetes-driven atherosclerosis: updated mechanistic insights and novel therapeutic strategies. Int J Mol Sci. 10.3390/ijms26052196 10.3390/ijms26052196 PMC11900163 40076813

[CR17] Kim YJ, Park S, Yi W, Yu KS, Kim TH, Oh TJ, Choi J, Cho YM (2014) Seasonal variation in hemoglobin a1c in Korean patients with type 2 diabetes mellitus. J Korean Med Sci 29(4):550–555. 10.3346/jkms.2014.29.4.550 24753703 10.3346/jkms.2014.29.4.550 PMC3991799

[CR18] Kim JT, Lee JS, Kim BJ, Kang J, Lee KJ, Park JM, Kang K, Lee SJ, Kim JG, Cha JK, Kim DH, Park TH, Lee KB, Lee J, Hong KS, Cho YJ, Park HK, Lee BC, Yu KH, Oh MS, Kim DE, Choi JC, Kwon JH, Kim WJ, Shin DI, Yum KS, Sohn SI, Hong JH, Lee SH, Park MS, Choi KH, Ryu WS, Lee J, Saver JL, Bae HJ (2023) Admission hyperglycemia, stroke subtypes, outcomes in acute ischemic stroke. Diabetes Res Clin Pract 196:110257. 10.1016/j.diabres.2023.110257 36642337 10.1016/j.diabres.2023.110257

[CR19] Kurtz P, Bastos LS, Aguilar S, Hamacher S, Bozza FA (2021) Effect of seasonal and temperature variation on hospitalizations for stroke over a 10-year period in Brazil. Int J Stroke 16(4):406–410. 10.1177/1747493020947333 32752950 10.1177/1747493020947333

[CR20] Lattanzi S, Bartolini M, Provinciali L, Silvestrini M (2016) Glycosylated hemoglobin and functional outcome after acute ischemic stroke. J Stroke Cerebrovasc Dis 25(7):1786–1791. 10.1016/j.jstrokecerebrovasdis.2016.03.018 27103269 10.1016/j.jstrokecerebrovasdis.2016.03.018

[CR21] Li S, Zhou Y, Williams G, Jaakkola JJ, Ou C, Chen S, Yao T, Qin T, Wu S, Guo Y (2016) Seasonality and temperature effects on fasting plasma glucose: a population-based longitudinal study in China. Diabetes Metab 42(4):267–275. 10.1016/j.diabet.2016.01.002 26851820 10.1016/j.diabet.2016.01.002

[CR22] Liang WW (2007) Seasonal changes in preprandial glucose, A1C, and blood pressure in diabetic patients. Diabetes Care 30(10):2501–2502. 10.2337/dc07-0597 17586743 10.2337/dc07-0597

[CR23] Luo J, He G, Xu Y, Chen Z, Xu X, Peng J, Chen S, Hu J, Ji G, Liu T, Zeng W, Li X, Xiao J, Guo L, He Q, Ma W (2021) The relationship between ambient temperature and fasting plasma glucose, temperature-adjusted type 2 diabetes prevalence and control rate: a series of cross-sectional studies in Guangdong Province, China. BMC Public Health 21(1):1534. 10.1186/s12889-021-11563-5 34380442 10.1186/s12889-021-11563-5 PMC8356456

[CR24] Mosenzon O, Cheng AY, Rabinstein AA, Sacco S (2023) Diabetes and stroke: what are the connections? J Stroke 25(1):26–38. 10.5853/jos.2022.02306 36592968 10.5853/jos.2022.02306 PMC9911852

[CR25] Narita K, Kario K (2023) Seasonal variation in blood pressure and its impact on target organ damage and cardiovascular disease incidence. Hypertens Research: Official J Japanese Soc Hypertens 46(7):1710–1711. 10.1038/s41440-023-01289-9 10.1038/s41440-023-01289-9 37106045

[CR26] Nicolo M, Boullata JI (2019) Serum 25OHD concentration as a predictor of haemoglobin A1c among adults living in the USA: NHANES 2003 to 2010. BMJ Nutr Prev Health 2(1):35–38. 10.1136/bmjnph-2019-000029 10.1136/bmjnph-2019-000029 PMC7664494 33235955

[CR27] Pereira MT, Lira D, Bacelar C, Oliveira JC, de Carvalho AC (2015) Seasonal variation of haemoglobin A1c in a Portuguese adult population. Arch Endocrinol Metab 59(3):231–235. 10.1590/2359-3997000000043 26154091 10.1590/2359-3997000000043

[CR28] Raphael A, Friger M, Biderman A (2021) Seasonal variations in HbA1c among type 2 diabetes patients on a semi-arid climate between the years 2005–2015. Prim Care Diabetes 15(3):502–506. 10.1016/j.pcd.2020.11.013 33309124 10.1016/j.pcd.2020.11.013

[CR29] Sakamoto M, Matsutani D, Minato S, Tsujimoto Y, Kayama Y, Takeda N, Ichikawa S, Horiuchi R, Utsunomiya K, Nishikawa M (2019) Seasonal variations in the achievement of guideline targets for HbA1c, blood pressure, and cholesterol among patients with type 2 diabetes: a nationwide population-based study (ABC study: JDDM49). Diabetes Care 42(5):816–823. 10.2337/dc18-1953 30739885 10.2337/dc18-1953

[CR30] Shi X, Yang S, Guo C, Sun W, Song J, Fan S, Yang J, Yue C, Huang J, Li L, Tian Y, Ma J, Xu X, Wang Z, Kong W, Ye D, Peng Z, Li F, Zi W (2025) Impact of stress hyperglycemia on outcomes in patients with large ischemic stroke. J Neurointerv Surg. 10.1136/jnis-2024-021899 10.1136/jnis-2024-021899 39299744

[CR31] Takai M, Ishikawa M, Maeda H, Kubota A, Iemitsu K, Umezawa S, Kawata T, Takuma T, Takeda H, Tanaka K, Machimura H, Minagawa F, Mokubo A, Motomiya T, Kanamori A, Matsuba I (2020) A study of seasonal variation in the effect of add-on sitagliptin on blood glucose control in insulin-treated patients with type 2 diabetes. J Clin Med Res 12(3):200–208. 10.14740/jocmr4103 32231757 10.14740/jocmr4103 PMC7092762

[CR32] Tseng CL, Brimacombe M, Xie M, Rajan M, Wang H, Kolassa J, Crystal S, Chen TC, Pogach L, Safford M (2005) Seasonal patterns in monthly hemoglobin A1c values. Am J Epidemiol 161(6):565–574. 10.1093/aje/kwi071 15746473 10.1093/aje/kwi071

[CR33] Vallianou NG, Geladari EV, Kounatidis D, Geladari CV, Stratigou T, Dourakis SP, Andreadis EA, Dalamaga M (2021) Diabetes mellitus in the era of climate change. Diabetes Metab 47(4):101205. 10.1016/j.diabet.2020.10.003 33127474 10.1016/j.diabet.2020.10.003

[CR34] Xue J, Liu P, Xia X, Qi X, Han S, Wang L, Li X (2023) Seasonal variation in neurological severity and clinical outcomes in ischemic stroke patients - a 9-year study of 5,238 patients. Circulation Journal: Official J Japanese Circulation Soc 87(9):1187–1195. 10.1253/circj.CJ-22-0801 10.1253/circj.CJ-22-0801 37032070

[CR35] Zhang Y, Tong M, Wang B, Shi Z, Wang P, Li L, Ning Y, Lu T (2021) Geographic, gender, and seasonal variation of diabetes: a nationwide study with 1.4 million participants. J Clin Endocrinol Metab 106(12):e4981–e4992. 10.1210/clinem/dgab543 34314489 10.1210/clinem/dgab543

[CR36] Zhang Y, Yin X, Liu T, Ji W, Wang G (2024) Association between the stress hyperglycemia ratio and mortality in patients with acute ischemic stroke. Sci Rep 14(1):20962. 10.1038/s41598-024-71778-5 39251650 10.1038/s41598-024-71778-5 PMC11385565

