# Supplementary material for: Effects of seasonal temperature variability on glucose levels in acute ischemic stroke patients
Source: Int J Biometeorol. 2026 Jan 5;70(1):12. doi: 10.1007/s00484-025-03103-2 (PMC12769608; doi:10.1007/s00484-025-03103-2)
Supplement: Supplementary file 1 — Supplementary Material 1 (PDF 1.65 MB) [file 484_2025_3103_MOESM1_ESM.pdf]

SUPPLEMENTAL MATERIAL

Graphical abstract

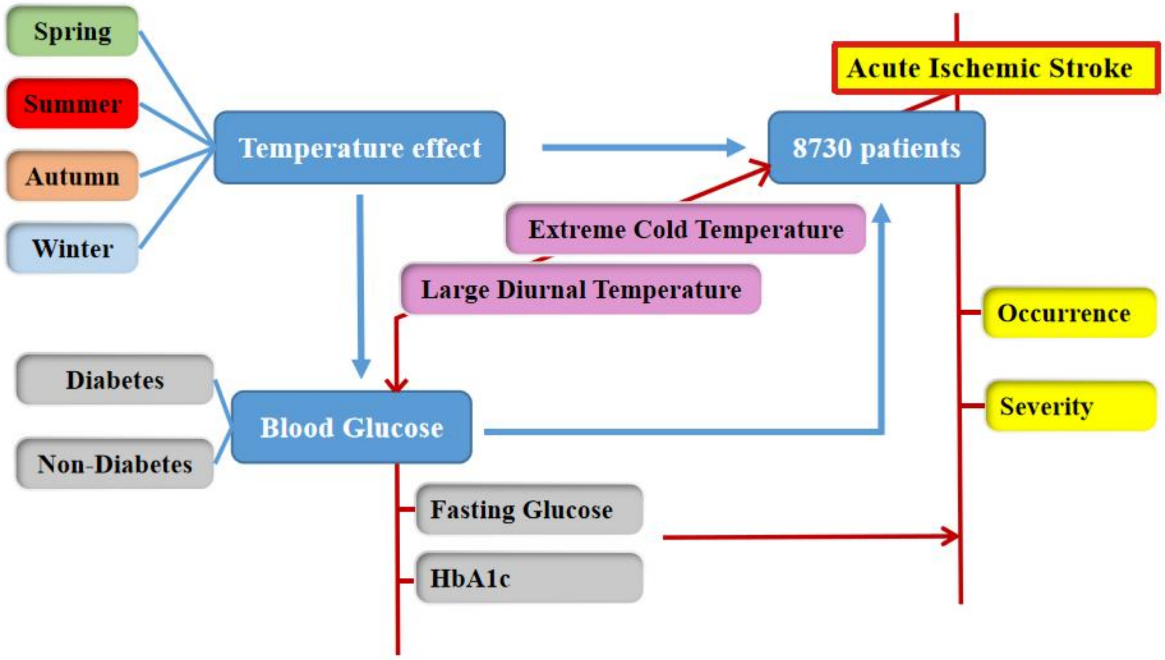

Supplemental Figure I. The geographical location of our hospital.

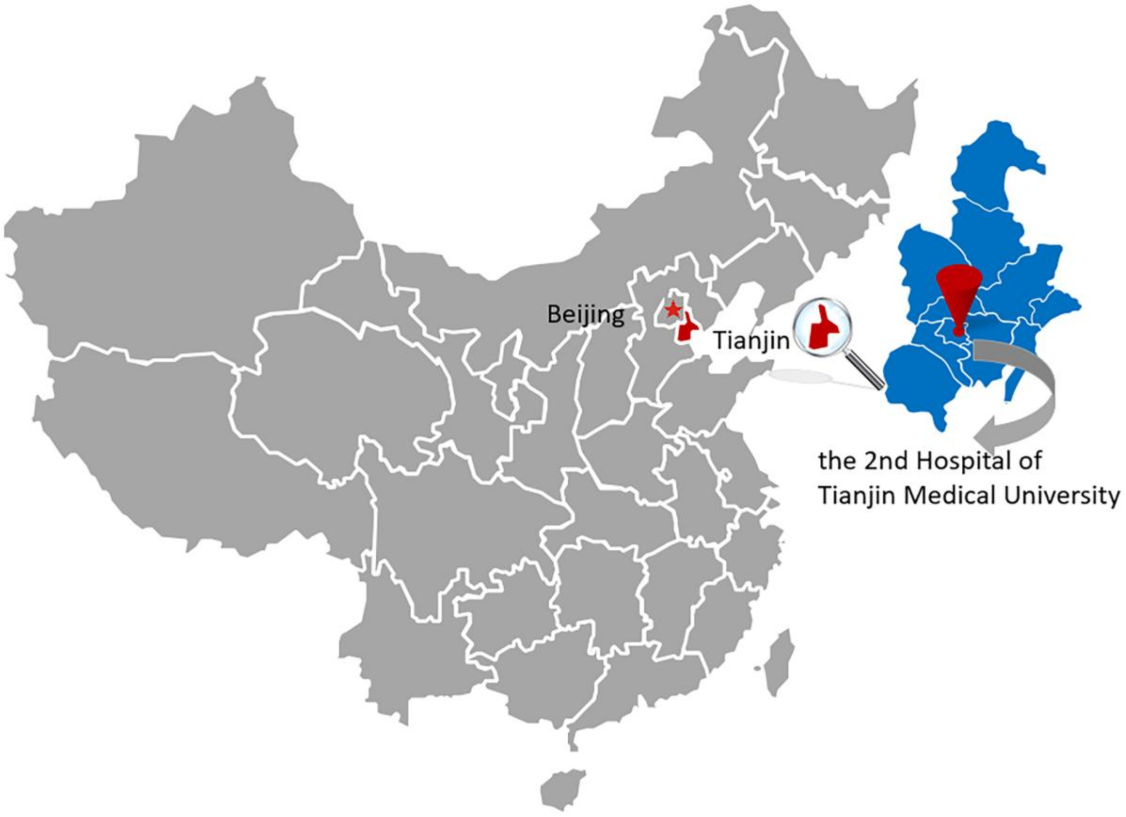

Supplemental Figure II. Monthly mean temperature and the incidence of acute ischemic stroke.

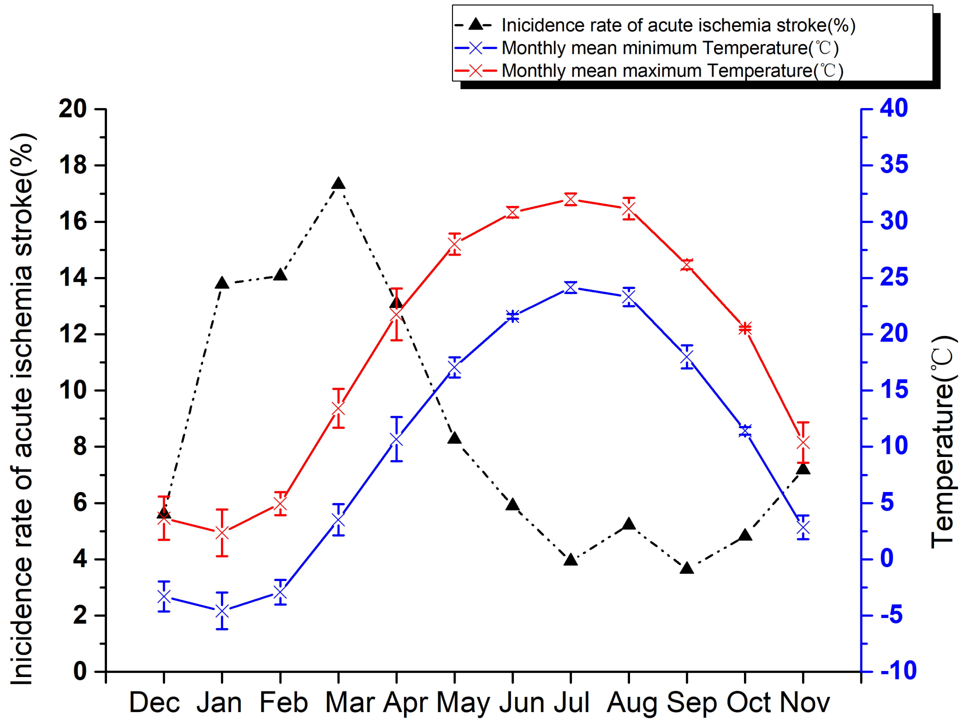

Supplemental Figure III. The seasonal change in levels of HbA1c in patients with diabetes.

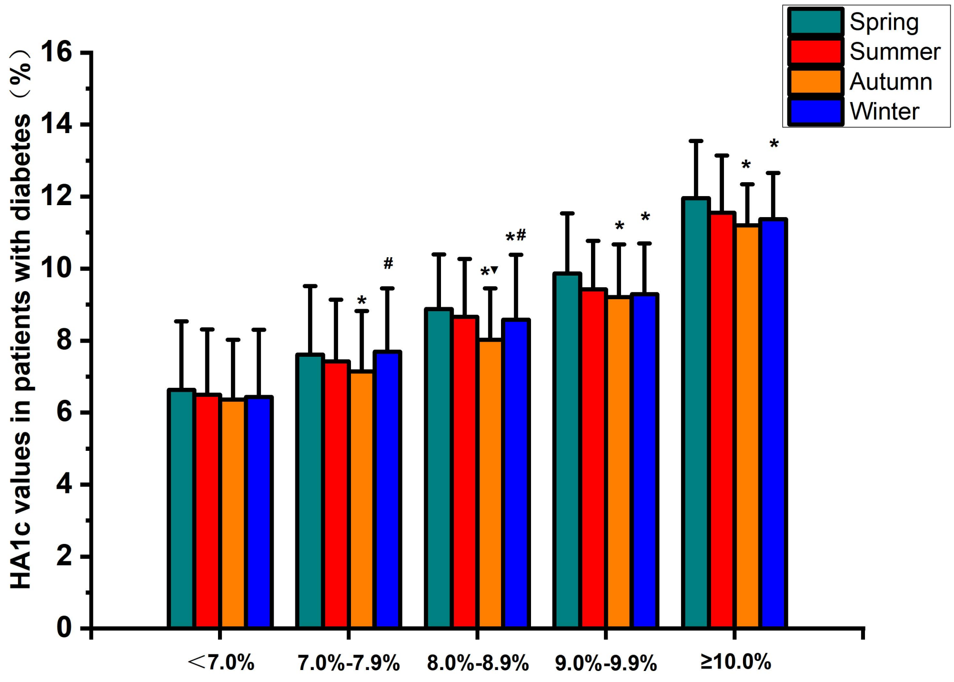

* $P < 0.05$ vs spring; ▼$P < 0.05$ vs summer; # $P < 0.05$ vs autumn.
